# Narrowing Yield Gaps and Enhancing Nitrogen Utilization for Summer Maize (*Zea mays L)* by Combining the Effects of Varying Nitrogen Fertilizer Input and Planting Density in DSSAT Simulations

**DOI:** 10.3389/fpls.2020.560466

**Published:** 2020-11-16

**Authors:** Hao Ren, Zhenhai Li, Yi Cheng, Jibo Zhang, Peng Liu, Rongfa Li, Qinglong Yang, Shuting Dong, Jiwang Zhang, Bin Zhao

**Affiliations:** ^1^State Key Laboratory of Crop Biology, College of Agronomy, Shandong Agricultural University, Tai’an, China; ^2^Beijing Research Center for Information Technology in Agriculture, Beijing Academy of Agriculture and Forestry Sciences, Beijing, China; ^3^Shandong Climate Center, Jinan, China

**Keywords:** DSSAT, nitrogen fertilizer, planting density, yield gap, NPFP

## Abstract

In China, the most common grain crop is maize (*Zea mays*). The increasing pressure to meet the food demands of its growing population has pushed Chinese maize farmers toward an excessive use of chemical fertilizers, a practice which ultimately leads to a massive waste of resources and widespread environmental pollution. As a result, increasing the yield and improving the nitrogen (N) use efficiency of maize has become a critical issue for agriculture in China. This study, which analyzes the combined data from a simulation carried out using the Decision Support System for Agrotechnology Transfer (DSSAT), a field experiment, and a household survey, explored the effectiveness of several approaches aimed at narrowing the maize yield gap and improving the N utilization efficiency in the Huang-Huai-Hai Plain (HHHP), the most important area for the production of summer maize in China. The various approaches we studied deploy different methods for the integrated management of N fertilizer input and the planting density. The study produced the following results: (1) For the simulated and actual maize yields, the root mean square error (RMSE), the normalized root mean squared errors (NRMSE) and the index of agreement (d) were 1,171 (kg ha^–1^), 12% and 0.84, respectively. These results show that the model is viable for the experiment included in the study; (2) The potential yield was 15.58 t ha^–1^, and the yields achieved by the super-high-yield cultivation pattern (SH), the optimized nutrient and density management pattern (ONM), the simulated farmer’s practice cultivation pattern (FP) and actual farmer’s practice (AFP) were 11.43, 11.06, 10.33, and 7.95 t ha^–1^, respectively. The yield gaps associated with the different yield levels were large; (3) For summer maize, the high yield and a high N partial factor productivity (NPFP) was found when applying a planting density of 9 plants m^–2^ and an N application amount of 246 kg ha^–1^. These results suggest that the maximum yield that can actually be achieved by optimizing the N application and planting density is less than 73% of the potential yield. This implies in turn that in order to further narrow the observed yield gaps, other factors, such as irrigation, sowing dates and pest control need to be considered.

## Introduction

Between 2000 and 2050, the global maize production is expected to grow by more than 450 million tons in order to meet the demands posed by population growth and improving living standards (Hubert et al., 2010). In 2017, China produced 2.59 million tons of maize, which accounted for 22.84% of the global maize production (FAO, 2018). The HHHP is China’s largest summer maize growing region: it accounts for 35% of its total maize planting area and 30% of its total maize production (National Bureau of Statistics, 2018). Increasing the maize yield in the HHHP is of great importance to China’s food security, and indeed that of the world. In the HHHP, the highest maize yield was 21,030 kg ha^–1^ (Li and Wang, 2010), whereas the average maize yield was only 5,881 kg ha^–1^ (National Bureau of Statistics, 2018), which points to a huge potential for increasing the yield.

Raising the potential crop yield is difficult to achieve in a short time (Rincent et al., 2014; Li et al., 2017; Zhang H. et al., 2018; Simkin et al., 2019), and therefore a substantial increase in current yields can only be achieved by narrowing the yield gap (Wang et al., 2014). Typically, the yield gap is defined as the difference between the average yield achieved by farmers in a given area over a certain period of time and the estimated reference yield (usually referred to as the potential yield or the water limit yield) (Maria Carolina et al., 2018; Agus et al., 2019; Marloes et al., 2019). The potential yield can be defined and measured in a number of ways, for example through using crop growth models, by conducting maximum yield trials, or by measuring the maximum yield achieved by farming households (Van Ittersum and Rabbinge, 1997; Lobell et al., 2009; Marloes et al., 2019). Currently, China’s actual maize yield is only 58% of the average potential yield of 13,875 kg ha^–1^, which makes for a yield gap of 42% (Global Yield Gap Atlas, 2019). This yield gap is the result of farmers not using suitable cultivars and production techniques. Narrowing the yield gap is a logical and modern strategy that has been touted as a solution to the world’s need to increase global food production (Lobell et al., 2009; Liu et al., 2016; Marloes et al., 2019). Several approaches to narrowing the yield gap have been studied. Zhang J. et al. (2019) reported that improving soil properties could reduce the maize yield gap. Cui et al. (2013) reported that effective N management could narrow the maize yield gap while reducing greenhouse gas emissions. All studies agree that quantifying the potential yield and the yield gap for maize could help to reveal the factors that limit the yield, and ultimately lead to suggestions for technical management measures to narrow the existing yield gap (Wang et al., 2014; Liu et al., 2016; Agus et al., 2019; Marloes et al., 2019).

Current research focuses on how to reduce the yield gap in a sustainable way. Over the past century, the availability of a reliable fertilizer supply has enabled farmers to greatly increase the crop yield per unit land area, and meet the food demands of a growing population (Foley et al., 2011). In order to increase their crop yield, some farmers overuse chemical fertilizers, which has caused an explosive increase of N fertilizer in China (FAO, 2018). Moreover, a large amount of resources is wasted, which is not conducive to the sustainable development of agriculture (Zhang et al., 2015). Therefore, researchers carried out several studies aimed at assessing the N nutrition status of crops and improving the N utilization efficiency in maize (Zhang et al., 2015; Zhang Y. et al., 2018; Li Z. H. et al., 2019). In one example, the application of N was carried out in stages or delayed (Guo et al., 2018), in another organic fertilizer and inorganic fertilizer were used together (Yang et al., 2019). Other lines of research found that a suitable planting density is another fundamental factor to be considered for achieving a high maize yield (Luo et al., 2020). Additional studies showed that integrating the management of the N fertilizer input and the planting density can be an important means to improve crop yield and N utilization efficiency (Wei et al., 2017). However, the planting densities used by different maize cultivators vary greatly in different ecological areas (Solomon et al., 2017), so it is necessary to determine both the appropriate N management method and the optimal planting density for each of those areas, notwithstanding the fact that the agronomic experiments needed to achieve this consume a lot of time and resources.

Crop growth models are agronomic decision systems recognized by scientists. By simulating probable outcomes of crop management strategies, they can be used to rapidly appraise new crops, products, and practices and qualify or disqualify them for adoption, so as to save a lot of the time and labor cost associated with experiments, making them important tools for agricultural technology research (Van Diepen et al., 1989; Dzotsi et al., 2010; Liu et al., 2016; Setiyono et al., 2019; Saddique et al., 2020). The DSSAT model is the most commonly used model package (Yakoub et al., 2017) to characterize the growth, development, yield, irrigation and N uptake of multiple crop species (Boote et al., 2017; Hoogenboom et al., 2019; Qu et al., 2019; Zhang D. et al., 2019). Examples of successful DSSAT deployments abound in the literature. Malik et al. (2019) reported that improved irrigation and N management practices as suggested by the DSSAT-CERES-maize model could improve maize yield and N use efficiency under Mediterranean climate conditions. Kadigi et al. (2020) used DSSAT to evaluate the economic feasibility of an improved planting density (used to realize an optimized plant population) and better N-fertilizer crop management practices for increasing maize net returns in semi-arid and sub-humid agro-ecological zones in the Wami River sub-Basin in Tanzania. Finally, Banger et al. (2018) used the DSSAT-CERES maize model to analyze the impact of N management on maize yield in the Midwest region of the U.S.

Conceiving production methods that balance increased production with more efficient N use is a major challenge. Most previous studies in this area have focused on single-factor agronomic management (Osmond et al., 2015; Yang et al., 2019), with a few studies combining N fertilization and planting density. Moreover, some studies only rely on a single experiment (Guo et al., 2016; Zhang Y. et al., 2018; Luo et al., 2020) or a single model simulation (Liu et al., 2012; Cheng et al., 2015; Malik et al., 2019), and fail to investigate the actual production levels achieved by farmers. In this paper, we combined a DSSAT model simulation with a field experiment and a peasant household survey to analyze the effects of integrated agronomic management on summer maize yield and N fertilizer utilization. The objectives of the research presented in this paper are (1) Using DSSAT to calibrate and validate the characteristics of summer maize in the HHHP of China, (2) Quantifying the yield gap for several agronomic management mode with different yield levels, and (3) Determining the optimal fertilizer application and planting density management practices under environmentally sustainable conditions.

## Materials and Methods

### Field Experiment

#### Sites and Experimental Conditions

The experiments were conducted from 2012 to 2016 at the Wenkou Experimental Station, Shandong Agricultural University, Taian, Shandong Province, China (35°58′10 N, 117°03′30 E and 178 m). The soil at the experimental field consists of sandy loam (Typic Hapli-udic Argosols); its physical and chemical properties are shown in Table 1.

**TABLE 1:** Physical and chemical properties of the experimental plot used in model evaluation and application.

**Depth**	**SOC (%)**	**SAP (mg kg^–1^)**	**PH in water**	**Total N (%)**	**Clay (%)**	**Silt (%)**	**Sand (%)**	**BD (g cm^–3^)**	**LL (cm^3^ cm^–3^)**	**DUL (cm^3^ cm^–3^)**	**SAT (cm^3^ cm^–3^)**	**CEC (cmol kg^–1^)**
0–20	2.17	95.03	5.36	0.16	28.5	32.4	39.1	1.32	0.14	0.29	0.44	10.6
20–40	1.35	15.92	6.37	0.06	23.0	30.2	46.8	1.39	0.09	0.26	0.35	10.8
40–60	0.61	3.40	6.84	0.04	25.7	25.9	48.4	1.45	0.09	0.24	0.32	12.3
60–80	0.50	4.73	7.32	0.03	26.1	30.6	43.3	1.44	0.10	0.26	0.34	12.7
80–100	1.05	6.27	6.99	0.03	25.9	33.1	41.0	1.35	0.11	0.27	0.37	14.7

*SOC, soil organic carbon; SAP, Soil Available Phosphorus; LL, lower limit; DUL, drained upper limit; SAT, saturation; SRGF, relative root distribution; BD, bulk density.*

#### Treatments and Experimental Design

All on-farm experiments followed a standardized experimental protocol. We set up three experimental treatments and defined the following three corresponding yield levels: SH, defined as the maximum yield under local climatic conditions with unlimited supply of nutrients and water; ONM, defined as the yield obtained after optimizing the fertilization program and planting density; and FP, the yield achieved by simulating the farmers’ management practices at the experimental station.

The experiment deployed a randomized block design with three treatments and four replications (Table 2). Each plot was 180 m^2^. Plots were separated by 30-cm-wide earthen dams to allow flood irrigation of each plot. Adequate irrigation was provided as required by the momentary growth rate of the maize. The experimental blocks were separated by 1-m walkways. N, P, and K were provided as urea (containing 46% N), superphosphate (containing 12% P_2_O_5_), and potassium chloride (containing 60% K_2_O), respectively. As organic fertilizer, we used chicken manure, which contained 315 g kg^–1^ organic carbon (dry basis), 32.3 g kg^–1^ P_2_O_5_, 30.4 g kg^–1^ K_2_O, and had a C/N of 11.3.

**TABLE 2:** Nutrient and density management measures at different management levels.

**Treatment**	**planting density (plant m^–2^)**	**Nutrient type**	**Nutrient input (kg ha^–1^)**	**Fertilization period and fertilization ratio (%)**				
				**Before sowing**	**6-leaf stage**	**12-leaf stage**	**Anthesis**	**One week after anthesis**
SH	9	N	450	10	20	30	20	20
		P_2_O_5_	270	100				
		K_2_O	450	50	50			
		Chicken manure	15000	100				
ONM	8.25	N	360	10	20	50		20
		P_2_O_5_	225	100				
		K_2_O	405	50	50			
FP	6	N	322.5	50		50		
		P_2_O_5_	112.5	48	52			
		K_2_O	195	20	80			
AFP	6	N	330 ± 105	50		50		
		P_2_O_5_	278 ± 84	100				
		K_2_O	275 ± 73	100				

#### Agronomic Management and Measurements

**FIGURE 1:**
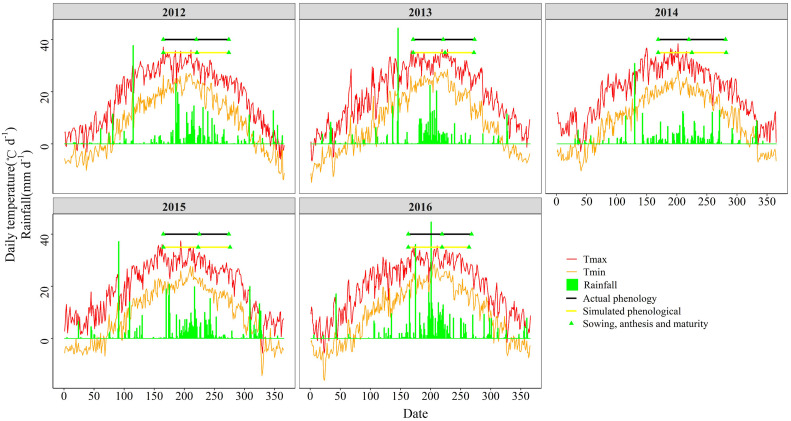
Meteorological data and actual and simulated phenological periods of summer maize from 2012 to 2016.

The experimental fields were managed by collaborating farmers, under the guidance of the researchers. The maize variety Zhengdan 958 (Zheng58 × Chang 7-2, ZD958), which is commonly planted in northern China, served as the experimental variety. The 5-year sowing and harvesting times are shown in Figure 1. Pest and weed control followed local high-yield practices. Physical and chemical data for the soil were obtained before planting. The phenological stage was accurately recorded: six plants from each replication were sampled at the following six growth stages: 9th leaf stage, 12th leaf stage, anthesis stage, milk stage, dent stage and maturity stage. To measure the biomass, all samples were heat-treated at 105°C for 30 min, and then dried at 70°C until the weight remained constant, after which they were weighed. At the same time, the leaf area of the maize was measured by leaf area meter (LI-3100C, LI-COR) and the leaf area index (LAI) was calculated. To determine the yield and its component measurements, all ears from 9 m^2^ at the center of the plots were harvested and the number of harvested plants was counted. For this study, the dry grain yield was used as the grain yield.

#### Actual Farmer’s Practice

The AFP yield was determined by a survey of 281 plots of agricultural land near the experimental site. The survey, which was carried out in 2016, asked farmers to provide the following information about their summer maize crop: yield, maize varieties planted, planting density, and the amount of fertilizer they applied (Table 2). Generally, the farmers irrigated their fields once after sowing and once at the 9-leaf stage.

### DSSAT Model

#### Model Description and Fundamental Data Set

DSSAT (version 4.7) (Hoogenboom et al., 2019) has been tested and applied extensively in China (Liu et al., 2012; Li et al., 2015; Zhang D. et al., 2019). It can be used to simulate the phenology, biomass and yield of maize. Crop simulation models simulate the growth, development, and yield of a specific crop as a function of a set of dynamic parameters characterizing the soil, the crop, and the atmosphere. The parameters comprise soil data, crop management data, and meteorological data. For each soil horizon, the soil data, including a number of physical indicators (soil texture, permanent wilting point, field capacity, volumetric water content at saturation and bulk density), and chemical indicators (soil organic carbon, inorganic N, and pH), were measured through ground investigations (Table 1). The crop management data used in the model (for example seeding time, irrigation parameters, planting density, and fertilizer input) follow the experimental parameters described in section “Field Experiment.” The meteorological data, including the precipitation, solar radiation, and minimum and maximum air temperature, were acquired from the European Centre for Medium-Range Weather Forecasts (Figure 1).

#### Model Parameter Calibration

The DSSAT-Maize model used for our study was calibrated using crop data for the 3 years from 2012 to 2014. We used a trial-and-error method to determine which maize crop parameters optimize the model’s performance (Li et al., 2015). The maize crop parameters were divided into three subsets: species-, ecotype-, and cultivar-specific (or genetic coefficients). The relevant subsets were saved with each DSSAT input file. For the species-specific parameters we used the DSSAT-Maize model’s default values for maize. The values for ecotype and cultivar-specific phenological parameters (Table 3), which are required for DSSAT-Maize, were obtained by fitting the model to the observed biomass, leaf area index (LAI), and yield, and to the dates of emergence, anthesis and maturity for the relevant experimental maize treatment (see Table 3 for the parameter description).

**TABLE 3:** DSSAT-CERES-maize model partial parameters meaning and calibration value of this study.

**Parameter type**	**Parameters**	**Parameter description**	**Values**
Regulate phenological periods	P1	Thermal time from seedling emergence to the end of the juvenile phase (expressed in degree days above a base temperature of 8°C) during which the plant is not responsive to changes in photoperiod.	270
	P2	Extent to which development (expressed as days) is delayed for each hour increase in photoperiod above the longest photoperiod at which development proceeds at a maximum rate (which is considered to be 12.5 h)	0.45
	P5	Thermal time from silking to physiological maturity (expressed in degree days above a base temperature of 8°C).	800
Regulate biomass	RUE	Radiation use efficiency, g plant dry matter/MJ PAR	4.3
Regulate LAI	PHINT	Phylochron interval; the interval in thermal time (degree days) between successive leaf tip appearances.	37.5
Regulate yield	G2	Maximum possible number of kernels per plant.	800
	G3	Kernel filling rate during the linear grain filling stage and under optimum conditions (mg/day).	6.9

### Statistical Analysis

#### Potential Yield, Rain-Fed Yield, Yield Gaps and N Partial Factor Productivity

According to its definition, the potential yield (YP), which is the yield of an adapted crop cultivar grown without limiting its water or nutrients supply, and without subjecting it to pests and diseases, is only affected by climatic conditions (note that the model does not include water and N constraints) (Evans and Fischer, 1999); The attainable yield (AY), which is defined as 80% of the potential yield, is difficult to exceed in regional field production, and the high water and fertilizer input it requires results in a reduced resource utilization efficiency (Lobell et al., 2009); The rain-fed yield is defined as the potential yield of rain-fed maize (limited only by water input by the model). Using these definitions along with the SH yield, the ONM yield, the FP yield, and the AFP yield (that is to say the yields achieved with the treatments defined in section “Materials and Methods”) several yield gaps can be defined:

(1) $${\mathrm{YG}}_{T}=\mathrm{YP}-\mathrm{AFP}$$

(2) $${\mathrm{YG}}_{1}=\mathrm{YP}-\mathrm{AY}$$

(3) $${\mathrm{YG}}_{2}=\mathrm{AY}-\mathrm{SH}$$

(4) $${\mathrm{YG}}_{3}=\mathrm{SH}-\mathrm{ONM}$$

(5) $${\mathrm{YG}}_{4}=\mathrm{ONM}-\mathrm{FP}$$

(6) $${\mathrm{YG}}_{5}=\mathrm{FP}-\mathrm{AFP}$$

To evaluate whether optimization measures lead to improvements in N utilization efficiency, we used the NPFP as an evaluation index:

(7) NPFP=Grain⁢yield/N⁢input

#### Model Performance Evaluation

The following statistical indexes were calculated to evaluate the performance of the DSSAT-maize model: RMSE, NRMSE, and d (Willmott, 1982). The calculation made use of the following equations:

(8) $$\mathrm{RMSE}=\sqrt{\frac{\sum_{\text{i}=1}^{\text{n}}{\left(S_{\text{i}}-{\text{O}}_{\text{i}}\right)}^{2}}{\text{n}}}$$

(9) $$\mathrm{NRMSE}=\sqrt{\frac{\sum_{\text{i}=1}^{\text{n}}{\left(S_{\text{i}}-O_{\text{i}}\right)}^{2}}{\text{n}}}\times \frac{100}{\bar{O}}$$

(10) $$d=1-\frac{\sum_{\text{i}=1}^{\text{n}}{\left(S_{\text{i}}-O_{\text{i}}\right)}^{2}}{\sum_{\text{i}=1}^{\text{n}}{\left(\left|{\text{S}}_{\text{i}}-\bar{\text{O}}\right|+\left|O_{\text{i}}-\bar{O}\right|\right)}^{2}}$$

In equations (8–10), S_i_ is the simulated data, O_i_ is the observed data, $\bar{O}$ is the average value of observed data, and *n* is the number of pairs of simulated and observed data.

Using the SPSS 13.0 software, an ANOVA analysis was performed on the differences between SH, ONM, and FP at the 0.05 level. Graphs were generated with the R for 3.0.0 software.

## Results

### Maize Phenology and Meteorological Data

From 2012 to 2016, the average time from sowing date to anthesis was 54.4 days, with a coefficient of variation of 7.4%, and the time from anthesis to maturity was 53 days, with a coefficient of variation (CV) of 9.3%. The average solar radiation during the growth period of summer maize was 2,857.59 MJ m^–2^, with a CV of 2.2%; the average effective accumulated temperature was 1,624.72°C, with a CV of 2.4%; and the average rainfall was 291.97 mm, with a CV of 25.1%. The precipitation varied considerably from year to year, mainly due to the fact that in 2014 the precipitation was unusually low (Figure 1). In order to guarantee that the maize could develop normally, the field management for this experiment was adapted to provide supplemental irrigation at times when the precipitation was insufficient.

### Model Calibration and Validation

**FIGURE 2:**
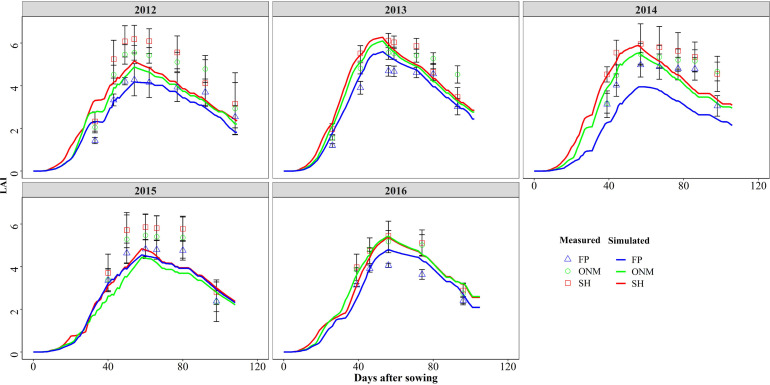
Comparisons between the measured and simulated values of LAI in three patterns during the summer maize growth seasons during 2012 and 2016.

**FIGURE 3:**
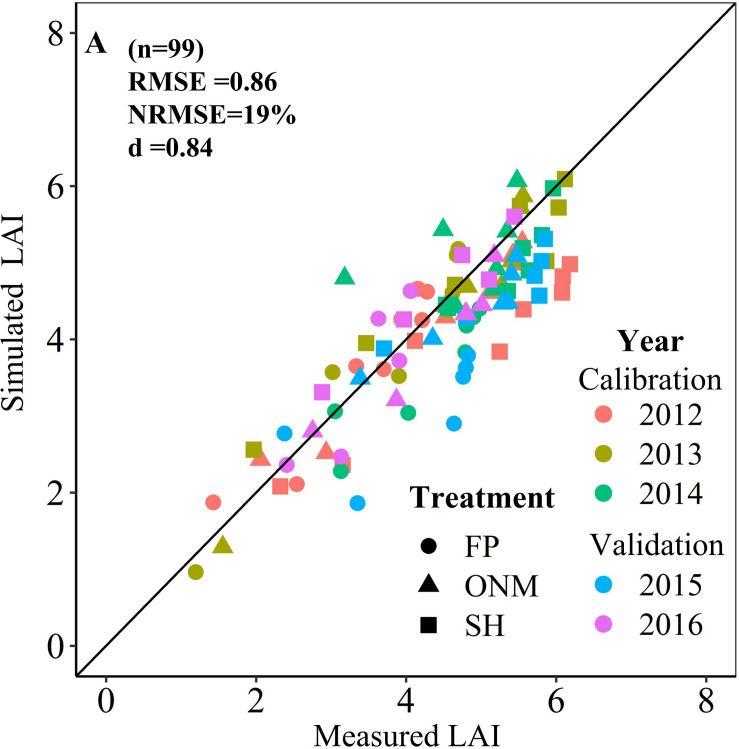
Relationship between simulated and measured LAI of summer maize in three patterns. The 2012–2014 data were used to calibrate the model, and the 2015–2016 data were used to validate the model.

**FIGURE 4:**
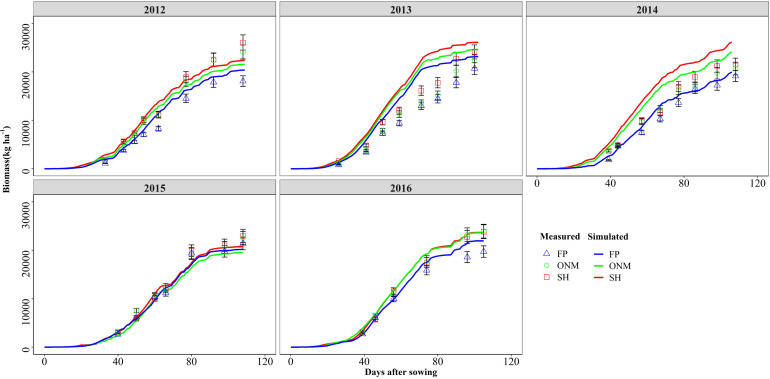
Comparisons between the measured and simulated values of biomass in three patterns during the summer maize growth seasons during 2012 and 2016.

**FIGURE 5:**
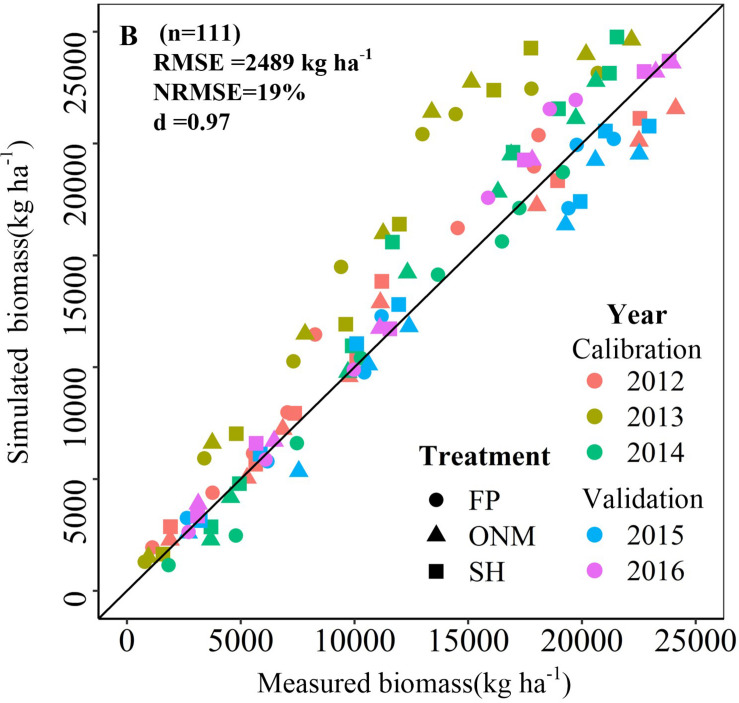
Relationship between simulated and measured biomass of summer maize in three patterns. The 2012–2014 data were used to calibrate the model, and the 2015–2016 data were used to validate the model.

**FIGURE 6:**
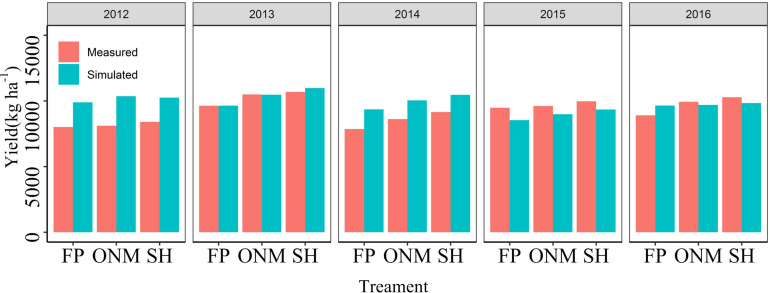
Comparisons between the measured and simulated values of yield in three patterns during the summer maize growth seasons during 2012 and 2016.

The DSSAT-Maize model was calibrated with the experimental data collected during 2012 and 2014, and validated with the experimental data collected during 2015 and 2016. The estimated cultivar coefficients for the Maize cultivar ZD958 are shown in Table 2. Over the 5 years covered by the simulation, the data indicate a close agreement between the simulated and observed values for anthesis, maturity, LAI, biomass and grain yield in maize. The model accurately simulated the number of days from seeding to anthesis and maturity with the RMSE of 2.47 days, the NRMSE of 1% and the *d*-value of 0.99 (Figure 1). Fitting the diagrams for the simulated LAI (Figure 2) and biomass (Figure 4) values to the measured values shows that the overall fit was good. The RMSE, NRMSE and d values for the LAI were 0.86, 19% and 0.84 (Figure 3), for the biomass they were 2,489 kg ha^–1^, 19% and 0.97 (Figure 5), and for the yield they were 1,171 kg ha^–1^, 12% and 0.84 (Figure 6), respectively. Overall, the validation showed that the DSSAT-Maize model could be deployed successfully to provide yield evaluations and decision diagnoses for this experimental station.

### Yield Potentials and Yield Gaps

**FIGURE 7:**
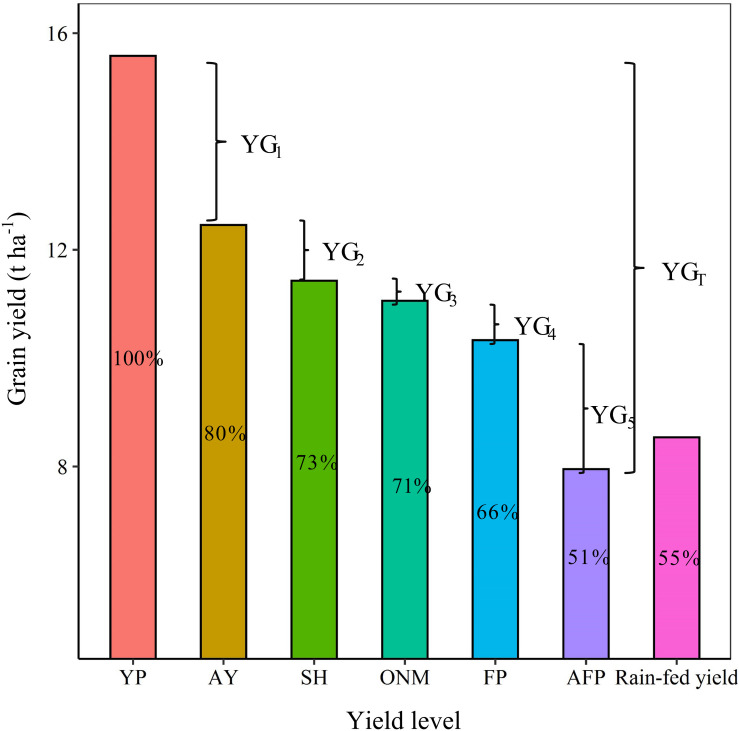
Summer maize yield at different grain yield levels. YP, yield potential; AY, attainable yield (80% of YP); SH, super-high-yield treatment; ONM, optimized nutrient and density management treatment; SFP, Simulated local farmer’s practice cultivation treatment; AFP, actual farmer’s yield (Household survey data); Rain-fed yield, potential of rain-fed maize yield (Only limited by water); YGi, yield gap between different yield levels (i is 1, 2, 3, 4, 5, and T). The percentage data in the bar chart is the ratio of different yield levels to the yield potential of summer maize.

The YP, AY, SH, ONM, FP, AFP, and Rain-fed yields were 15.58, 12.46, 11.43, 11.06, 10.33, 7.95, and 8.54 t ha^–1^, respectively. The YG_1_, YG_2_, YG_3_, YG_4_, YG_5_, and YG_T_ were 3.12, 1.03, 0.37, 0.73, 2.38, and 7.63 t ha^–1^, respectively. The SH, ONM, FP, AFP, and Rain-fed yield accounted for 73.39, 71.01, 66.31, 51.03, and 54.8 (%) of the YP, respectively (Figure 7). At the same time, the rain-fed potential only accounted for 54.8% of the YP, indicating that irrigation was an indispensable and important measure in the location under study.

### Simulated Yield Response to N Fertilizer Amount and Planting Density

**FIGURE 8:**
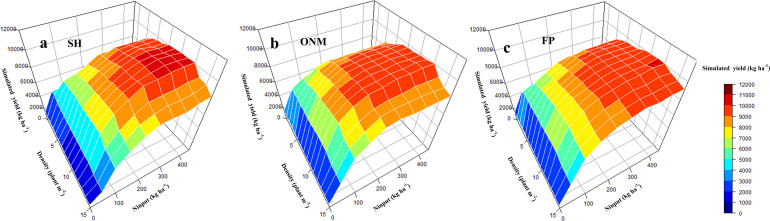
The response of simulated maize yield to nitrogen fertilizer and planting density. The figure above shows the simulated yield Response to different fertilization methods based on SH pattern **(a)**, ONM pattern **(b)**, and FP Pattern **(c)** respectively. SH, the ratio of organic fertilizer to nitrogen chemical fertilizer was 1:1, and base fertilizer and topdressing at the following growth stages: 6-leaf stage, 12-leaf stage, anthesis, and 1 week after the anthesis stage with a ratio of 1:2:3:2:2; ONM, basal fertilizer and topdressing at growth stages 6-leaf stage, 12-leaf stage, and 1 week after the anthesis stage with a ratio of 1:2:5:2 by nitrogen chemical fertilizer; FP, basal fertilizer and fertilizer dressed at the 12-leaf stag at a ratio of 1:1 by nitrogen chemical fertilizer.

**FIGURE 9:**
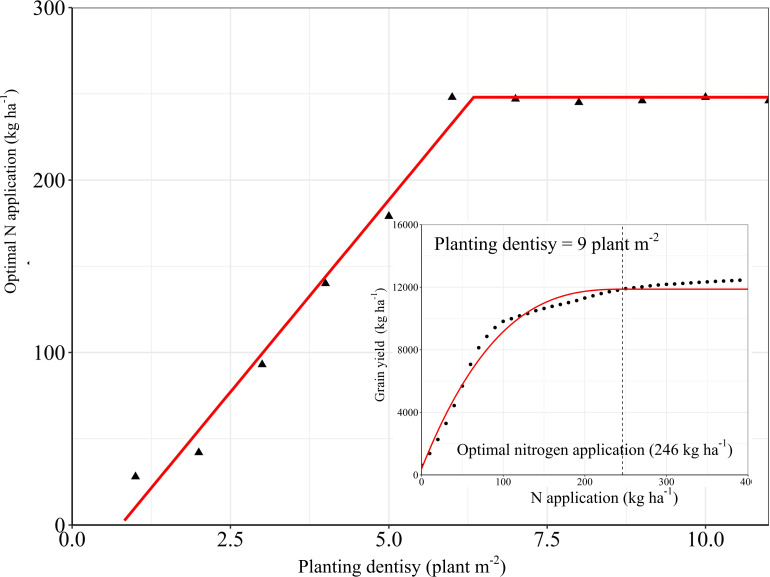
The response of optimal nitrogen application to planting density was simulated by DSSAT. The lower-right corner figure is the fitting analysis of the N application at the planting density was 9 plant m^–2^, and the optimal N application amount is 246 kg ha^–1^

The N fertilizer input and planting density are important agronomic measures to improve maize yield (Wei et al., 2017). The relationship between the N application amount and the grain yield is logarithmic. For the same planting density, an increase in N fertilizer input causes the yield to increase gradually, but at the same time the yield increase rate is seen to decrease gradually. The relationship between the planting density and the maize yield is polynomial. For the same N input, an increase in the planting density causes the yield to increase initially, but then decrease again, where the point at which the yield peaks depends on the N input (Figure 8). Fitting the yield response to the N fertilizer application and planting density showed that the optimal N application amount increases linearly as the planting density increases, until the latter reaches 6.2 plants m^–2^, after which the optimal N application amount remains constant. For a density of 9 plants m^–2^, the optimal N application amount was 246 kg ha^–1^ (Figure 9). For each of the three fertilization methods simulated by the DSSAT model, the yield response to the N fertilizer amount and the planting density was fundamentally consistent. Interannual climate variation mainly affected the upper limit of the yield and did not significantly affect the yield response to the N fertilizer amount and the planting density (Figure 8).

### NPFP Response to N Fertilizer Amount and Planting Density

**FIGURE 10:**
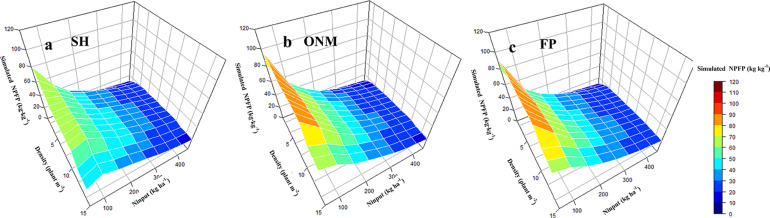
The response of simulated maize NPFP to nitrogen fertilizer and planting density. The figure above shows the simulated NPFP Response to different fertilization methods based on SH pattern **(a)**, ONM pattern **(b),** and FP Pattern **(c)** respectively. SH, the ratio of organic fertilizer to nitrogen chemical fertilizer was 1:1, and base fertilizer and topdressing at the following growth stages: 6-leaf stage, 12-leaf stage, anthesis, and 1 week after the anthesis stage with a ratio of 1:2:3:2:2; ONM, basal fertilizer and topdressing at growth stages 6-leaf stage, 12-leaf stage, and 1 week after the anthesis stage with a ratio of 1:2:5:2 by nitrogen chemical fertilizer; FP, basal fertilizer and fertilizer dressed at the 12-leaf stag at a ratio of 1:1 by nitrogen chemical fertilizer.

**FIGURE 11:**
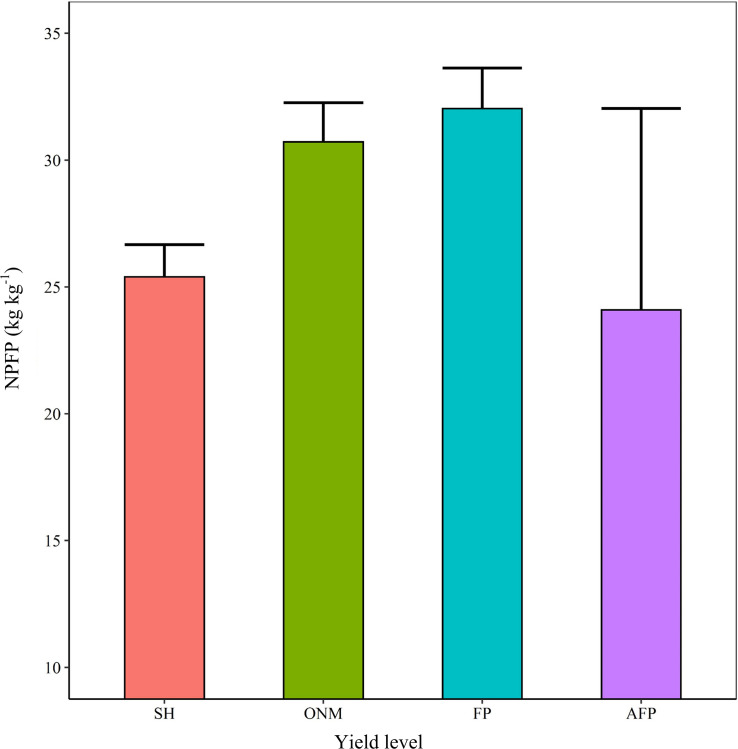
Summer maize nitrogen partial factor productivity (NPFP) at different yield levels.

Optimizing the N fertilizer amount and increasing the planting density were effective methods to improve the N use efficiency (Zhang D. et al., 2019). The relationship between the N application amount and the NPFP follows a power function. With increasing N input, the NPFP for maize decreased gradually at a decreasing rate. For the same N fertilizer input, an increase in the planting density caused the NPFP to increase initially, and then decrease again. Interactions between the N fertilizer input and the planting density had a significant effect on the NPFP. Figure 10 shows that for the optimal planting density of 9 plants m^–2^ and the optimal N fertilizer input of 247 kg ha^–1^, the simulated NPFP was 40.29 kg kg^–1^, which was 67.24% higher than the NPFP for AFP, and 25.90% higher than the NPFP for FP (Figure 11). As simulated by the DSSAT model, the rules for the NPFP response to N fertilizer and planting density are fundamentally consistent for all three fertilization methods. Interannual climate change mainly affected the upper limit of the NPFP and had little effect on the rules for the NPFP response to N and planting density (Figure 10).

## Discussion

### Model Simulation and Application

Crop models have been widely used to simulate and compare crop yields that are hard to study in the field directly. The DSSAT model has been proven to be applicable to spring maize (Liu et al., 2012; Li et al., 2015), but it has been used less frequently for summer maize growth simulations in China. As described in “Maize Phenology and Meteorological Data,” despite some deviations in the process used to simulate the LAI, the biomass and the yield of maize, the statistical analysis and evaluation of the data indicated that for this experiment the model provided a quite reliable simulation (Figures 1–5). This suggested that the DSSAT model could successfully simulate the summer maize production potential, which suggests that we can use it to make agricultural management decisions for our experimental sites. The DSSAT model has been widely used to provide an accurate basis for cultivation measures like N application, planting density and irrigation amount, and it has been improved constantly over the years (Amouzoua et al., 2019; Malik and Dechmi, 2019; Saddique et al., 2019). Nevertheless, we still found some deficiencies in the model when we used it, as described below.

Figure 6 shows that for 2012 and 2014, the measured yields were lower than the simulated yield, which could have been caused by rust diseases in the late growth period of the maize (Data not shown). Analogous to the studies mentioned before, the LAI increase and the aging exhibited by the model are also affected by soil moisture content (Çakir, 2004). For example, the model subroutine LAIS, which is used to calculate changes in the LAI, limits the potential rate by taking the soil water stress (both deficit and saturation), soil water stress factors, temperature, and growth rate reduction factor into account (Kraalingen and Van, 1995; Reynolds and Acock, 1997). The reason why the simulated LAI is low may be that the test site applies limited irrigation (Figure 3). Applying N fertilizer according to the rules of plant N requirement is an effective way to the increase maize yield and N utilization efficiency (Zhan et al., 2011; Perez, 2015; Osmond et al., 2015; Zhang et al., 2015). In this study, this fertilization method was adopted for the SH and ONM treatments, but the simulation results showed no significant difference between the yield response to the divided and the concentrated application of N, meaning they cannot be used to make effective recommendations for the application time and proportion of N (Figures 7, 8).

### Narrowing Yield Gaps Through Adaptation of Agricultural Practices

The purpose of this study was to identify and quantify the factors that restrict maize production in the HHHP and to assess the effectiveness of various integrated cultivation practices aimed at narrowing the yield gap caused by agronomic mismanagement. The results point at several significant opportunities to increase the maize yields in this area beyond their current levels. The survey data show that the AFP treatment achieved 51% of the potential yield and 93% of the rain-fed potential yield (Figure 7). Other studies have reported yield gaps that were not consistent with our findings. Liu et al. (2016) reported that farmers in Northeast China only achieved 36% of the potential yield of spring maize, while Li S. et al. (2019) believed that the summer maize yield achieved by farmers in North China reached 72% of the potential yield. The most likely explanation for the discrepancies between the findings of those studies and ours is the fact that the former concern geographical areas that are quite different from the area we studied. Furthermore, our study indicated that in areas where irrigation is used to grow summer maize, the main factor limiting yield increases is not a lack of water, but the application of a suboptimal combination of N fertilizer and planting density. Therefore, an agronomic management model for narrowing the yield gap should consider the integrated management of the N fertilizer amount and the planting density as the most effective means to increase the yield.

The attainable yield is the maximum yield that can be achieved at a field scale or at a regional scale (Li S. et al., 2019). Previous studies have shown that even with the best cultivation management techniques, it is difficult to achieve a crop yield that exceeds 80% of the biophysical ‘potential yield,’ even at a field scale (Lobell et al., 2009). Therefore, we define the attainable yield as 80% of the potential yield. Referring back to the yield gap definitions used in the statistical analysis YG_1_ is mainly affected by uncontrollable factors that cannot be completely matched, for example uncontrollable climate events and changes in crop management quality. SH, which is designed to provide an excessive nutrient supply at the optimum planting density, aims to achieve the maximum field-scale yield in the experimental area. YG_2_ is primarily limited by management factors other than nutrient input and planting density, such as sowing date, pests and diseases, or soil conditions. According to the simulation analysis, when the N fertilizer input exceeded 247 kg ha^–1^ (Figure 9), the effect of N fertilizer input on the yield increase was no longer significant. This indicates that the factors with the greatest influence on YG_3_ and YG_4_ are the N fertilizer application mode (application period) and the planting density. YG_5_ is the yield gap that is the most likely to be narrowed. Considering that FP adopts a fertilizer input and planting density similar to AFP, we can conclude that it is mainly caused by underutilization of agricultural technologies (including variety selection, irrigation management, and pest control). Therefore, any approach to reducing the yield gap should focus on improving the agrotechnical service provisions for low-yield farms.

### Improving Yield and NPFP by Adopting Better Agricultural Practices

Optimizing N fertilizer management practices is an important aspect of reducing production costs and environmental risks. Due to the diversity of fertilization methods and factors that influence their effectiveness, the optimal fertilizer application rate for crops in different cropping systems in different regions is subjected to large variations. Taking the economic benefits and the N utilization efficiency into account, a dynamic biogeochemical model for maize recommended an N application rate of 191 kg N ha^–1^ for three states in the Midwest region of the United States (Sela et al., 2018). Chen et al. (2011) reported that the optimal N application rate for maize as recommended by the soil crop management system was 237 kg N ha^–1^ in China; Zhang Y. et al. (2018) showed that the optimal N application rate for maize as suggested by the results of field trials conducted in the North China Plain was 240 kg N ha^–1^. Our study showed that the yield response to the N application amount is significantly reduced when it exceeds 246 kg N ha^–1^, and that continued N application would not only be ineffective for increasing the yield, but also lead to a reduced NPFP. This means that there is room for further optimization of the N management practices deployed in our ONM treatment. On the other hand, when applying an adaptive N condition, densification is an effective way to obtain a higher yield and a higher N utilization efficiency. However, once a density of 12 plants m^–2^ is reached, further densification is counter-effective, and decreases the yield (Figures 8, 10). It is worth noting that, although the light and temperature conditions of the test site meet the requirements for a planting density of 12 plants m^–2^, it is problematic to apply a planting density exceeding 9 plants m^–2^ due to the impact of high winds (Guo et al., 2018). The research conducted by Luo et al. (2020) in Northern China also verified our results. It is true that when applying a high planting density the upper yield limit is proportional to the N fertilizer rate. The optimal N application rate for the used planting density, however, is more or less constant for high planting densities, which enables farmers to treat the relationship between N fertilizer input and maize yield more rationally (Figure 9). In the near future, guiding and encouraging farmers to improve their fertilizer management and cultivation techniques in appropriate ways is essential for the sustainable development of agriculture, not only in China but around the world.

Our simulation analysis method is based on previous studies (Banger et al., 2018; Li S. et al., 2019; Malik and Dechmi, 2019; Malik et al., 2019). We did not adopt a long-term meteorological simulation; a lack of analyses quantifying the impact of climate change precludes a successful incorporation into any simulation. In order to make a better recommendation, we plan to conduct a multi-point verification of the conclusions from this experiment in the next round of research, and further analyze the relevant effects of different N fertilizer management measures (notably applying N fertilizer in stages, and applying organic and inorganic fertilizer together).

## Conclusion

This study found that the DSSAT model can accurately simulate the effect of cultivating measures on maize, and that the simulation results for maize phenology, LAI, biomass and yield accurately match the actual yield results.

The YG_T_ between AFP and YP in the HHHP was 7.63 t ha^–1^, which indicates a high potential for yield improvement. The results showed that the optimization of cultivation management measures, for example coordinating the N application amount and the planting density, could effectively increase the AFP yield by reducing the yield gap to 4.52 t ha^–1^. Adopting an N fertilizer rate of 246 kg ha^–1^ and a planting density of 9 plants m^–2^ are effective measure for improving the maize yield and the N use efficiency of summer maize cultivated in the HHHP.

## Data Availability Statement

The original contributions presented in the study are included in the article/supplementary material, further inquiries can be directed to the corresponding authors.

## Author Contributions

HR wrote the manuscript. ZL provided the technical support. YC, JZ, RL, and QY assisted the completion of test. PL designed the test plan. PL, SD, JZ, and BZ provided the testing equipment and environment. All the authors contributed to the article and approved the submitted version.

## Conflict of Interest

The authors declare that the research was conducted in the absence of any commercial or financial relationships that could be construed as a potential conflict of interest.
